# Supplementary material for: A probabilistic knowledge graph for target identification
Source: PLoS Comput Biol. 2024 Apr 5;20(4):e1011945. doi: 10.1371/journal.pcbi.1011945 (PMC11034645; doi:10.1371/journal.pcbi.1011945)
Supplement: S1 Text — Figure A, Tables A-N, and other supplementary information are included. Table A: Summary of the biological networks used in our performance evaluation process and their respective data sources. Table B: Summary of the new biological networks used in the applications of Progeni to predict the target candidates of human melanoma and colorectal cancer and their respective data sources. Table C: The supplementary results on the ablation studies in the cluster-wise cross validation test (mean ± standard deviation). The results where Progeni outperformed all control methods are presented in bold. Table D: The top 10 unobserved targets candidates predicted by Progeni for bronchitis. Table E: The top 10 unobserved targets candidates predicted by Progeni for pulmonary arterial hypertension (PAH). Table F: The top 10 unobserved targets candidates predicted by Progeni for liver neoplasms. Table G: Literature counts for the top 15 novel targets predicted by Progeni for melanoma. Table H: Literature counts for the top 15 novel targets predicted by Progeni for colorectal cancer. Table I: OD values for the B16F10 cells (melanoma) following shRNA knockdown. The results correspond to Fig 5A in the manuscript. Table J: OD values for the MC38 cells (CRC) following shRNA knockdown. The results correspond to Fig 6A in the manuscript. Table K: OD values for the B16F10 cells (melanoma) after 48h treatment with the HSP90AB1 inhibitor tanespimycin. The results correspond to Fig 5E in the manuscript. Table L: OD values for the B16F10 cells (melanoma) after 48h treatment with the HSP90AB1 inhibitor SNX-5422. The results correspond to Fig 5F in the manuscript. Table M: OD values for the MC38 cells (CRC) after 48h treatment with the ADRA2A inhibitor phentolamine. The results correspond to Fig 6E in the manuscript. Table N: OD values for the CT26 cells (CRC) after 48h treatment with the ADRA2A inhibitor phentolamine. The results correspond to Fig 6F in the manuscript. Fig A: (A)-(B), Survival cu [file pcbi.1011945.s001.pdf]

Supplementary Information for "A probabilistic knowledge graph for target identification"

Chang Liu[1,†], Kaimin Xiao[2,3,†], Cuinan Yu[4,†], Yipin Lei[1,†], Kangbo Lyu[1], Tingzhong Tian[1], Dan Zhao[1,*], Fengfeng Zhou[5,*], Haidong Tang[2,*], and Jianyang Zeng[6,7,8*]

[1]Institute for Interdisciplinary Information Sciences, Tsinghua University, Beijing, China.
[2]School of Pharmaceutical Sciences, Tsinghua University, Beijing, China.
[3]Joint Graduate Program of Peking-Tsinghua-NIBS, School of Life Sciences, Tsinghua University, Beijing, China.
[4]Machine Learning Department, Silexon AI Technology Co., Ltd., Nanjing, Jiangsu Province, China.
[5]Key Laboratory of Symbolic Computation and Knowledge Engineering of Ministry of Education, College of Computer Science and Technology, Jilin University, Changchun, Jilin Province, China.
[6]School of Engineering, Westlake University, Hangzhou, China
[7]Westlake Laboratory of Life Sciences and Biomedicine, Hangzhou, China
[8]Research Center for Industries of the Future and School of Engineering, Westlake University, Hangzhou, Zhejiang Province, China
[†]These authors contributed equally.
[*]Corresponding authors: zhaodan2018@tsinghua.edu.cn (DZ),
FengfengZhou@gmail.com (FZ),
hdtang@mail.tsinghua.edu.cn (HT),
zengjy@westlake.edu.cn (JZ).

1 Supplementary details on the biological network data

In our performance evaluation process, we compiled the input biological networks mainly from [1]. The details of the networks in this dataset are summarized below in Table A.

Relation type	Size	#Edges	Data source
Protein-protein interaction	$(1512, 1512)$	7363	HRPD database release 9 [2]
Drug-drug interaction	$(708, 708)$	10036	Drugbank 3.0 [3]
Drug-protein interaction	$(708, 1512)$	1932	
Drug-disease association	$(708, 5603)$	199214	Comparative Toxicogenomics Database [4]
Protein-disease association	$(1512, 5603)$	1596745	
Drug-side effect association	$(708, 4192)$	80164	SIDER database version 2 [5]

Table A: Summary of the biological networks used in our performance evaluation process and their respective data sources.

The computation of two additional biological networks, namely, the *drug-drug structure similarity* and *protein-protein sequence similarity* networks, are described in Methods of the main text.

In the applications of Progeni to identify the target candidates of human melanoma and colorectal cancer with experimental validation, we expanded and recompiled the network data from new sources to incorporate wider ranges of proteins and diseases. The network data coupled with literature evidence were again used to construct a new probabilistic knowledge graph, on which we retrained the Progeni model. The new network data are summarized in Table B:

Relation type	Size	#Edges	Data source
Protein-protein interaction	$(9045, 9045)$	611082	Decagon [6] (`http://snap.stanford.edu/decagon`)
Drug-drug interaction	$(227, 227)$	5748	MINER [7]
Drug-protein interaction	$(227, 9045)$	10920	Decagon [6], MINER [7]
Drug-disease association	$(227, 10111)$	93185	MINER [7]
Protein-disease association	$(9045, 10111)$	77429	DisGeNET [8]
Drug-side effect association	$(227, 8084)$	67606	Decagon [6]

Table B: Summary of the new biological networks used in the applications of Progeni to predict the target candidates of human melanoma and colorectal cancer and their respective data sources.

Here, the networks in MINER [7] were mainly based on the Drugbank 5.0 database [9] and the Comparative Toxicogenomics Database [4], and the networks in Decagon [6] were mainly based on the STITCH 5 database [10]. In addition, for the protein-disease association network, we used only the expert-curated data in DisGeNET [8], i.e., without incorporating those samples inferred from indirect evidence. We now describe the process of integrating these heterogeneous biological networks into the construction of the prob-KG. First, we retained only those diseases in DisGeNET [8] with corresponding MeSH/OMIM IDs. Next, among all protein-protein interactions from Decagon [6], we only retained those between proteins in DisGeNET. We then used the PubChem identifier exchange service (`http://pubchem.ncbi.nlm.nih.gov/idexchange`) to convert the drug identifiers adopted by Decagon and MINER to InChI identifiers, and stored their intersections as the *shared drugs*. The drug-drug interaction network was built by unioning the interactions observed in Decagon

and MINER between the shared drugs. We then retained the drug-disease associations in MINER with drugs from the list of shared drugs and diseases from DisGeNET. Finally, we kept the drug-side effect associations in Decagon with drugs from the list of shared drugs.

2 Baseline methods

In this section, we briefly describe the baseline methods used in the comparisons with Progeni during our performance evaluation process.

2.1 GTN

Graph transformer network (GTN) [11] is a state-of-the-art graph neural network (GNN) that has been shown to achieve excellent performance on several benchmark node-classification tasks on heterogeneous networks (HNs). GTN utilizes *meta-paths*, i.e., paths on an HN connected with edges of different types, based on which new adjacency matrices can be generated by multiplying the individual adjacency matrices along the segments of meta-paths. Instead of relying on the pre-defined meta-paths that generally require domain knowledge or manual selection, GTN learns the meta-paths by softly selecting adjacency matrices based on the attention mechanism.

Though GTN is a node classification model, we can easily reform it to perform target identification by appending a network reconstruction module similar to Eq. 5 and Eq. 8 in the main text:

$$\min \sum_{r} \|Z_a G_r H_r^T Z_b^T - A_r\|_2^2, \tag{S1}$$

where Z_a and Z_b stand for the node embedding matrices derived from Z (see Eq. 5 in [11]), a matrix representing all the learned feature embeddings of nodes, and A_r is the same as defined in Eq. 3 in the main text.

2.2 DTINet

DTINet [1] is a machine learning-based framework for drug-target interaction (DTI) prediction through integrating heterogeneous network information. DTINet employs random walk with restart (RWR) and diffusion component analysis (DCA), together with a final projection step as defined in Eq. 8 in the main text. To reform the original DTI prediction task for target identification, we only need to modify the RWR step in DTINet, which generates the diffusion states, i.e., latent representations, of relevant nodes. More Specifically, DTINet integrates all individual networks containing drugs and proteins (except for the drug-protein interaction network) to compute the diffusion states of drug and protein nodes, respectively. We modified this RWR step by integrating all networks containing diseases and proteins (except for the target-disease association network) to compute the diffusion states of the disease and protein nodes, respectively.

2.3 RGCN

Relational graph convolutional network (RGCN) [12] is a simple GNN designed for addressing the prediction tasks related to the highly multi-relational graphs. RGCN employs simple linear message transformations in the graph convolutional layers to learn the hidden feature representations of nodes. To perform target identification, we leveraged the same learning module as defined in Eq. 8 in the

main text, after obtaining the feature embeddings of nodes through the forward propagation module of RGCN.

2.4 HGT

Heterogeneous graph transformer (HGT) [13] is one of the state-of-the-art GNNs for modelling the Web-scale heterogeneous graphs. HGT adopts heterogeneous mutual attention [13] to aggregate information from neighbors. Briefly, in each iteration, query vectors are generated from the source node, while the key and value vectors are generated from the neighboring nodes. With the generated query, key, and value vectors, the attention mechanism is then employed to aggregate the neighborhood information. HGT additionally initiates a balanced neighborhood sampler, namely HGSampling [13], for large-scale training, which is not included in our implementation as only a relatively small number of nodes are in our HN used (see Methods in the main text). HGT learns the contextualized feature representations of nodes for the downstream tasks such as missing link prediction, and we use the same module as defined in Eq. 8 in the main text to perform target identification.

3 Supplementary results on the ablation studies in the cluster-wise cross-validation test

	Progeni_og	Progeni_rpw	Progeni_rp	Progeni_rw	Progeni
AUROC	0.810 ± 0.008	0.806 ± 0.007	0.808 ± 0.009	0.808 ± 0.007	$\mathbf{0.814 \pm 0.007}$
AUPR	0.501 ± 0.017	0.490 ± 0.013	0.502 ± 0.022	0.501 ± 0.014	$\mathbf{0.516 \pm 0.016}$

Table C: The supplementary results on the ablation studies in the cluster-wise cross validation test (mean $\pm$ standard deviation). The results where Progeni outperformed all control methods are presented in bold.

4 Top predictions for bronchitis, pulmonary arterial hypertension (PAH), and liver neoplasms

Protein Name	Uniprot ID	Prediction Score
CTNB1	P35222	0.711
CDK2	P24941	0.632
CTFR	P13569	0.587
APOA1	P02648	0.553
XDH	P47989	0.509
AGTR2	P50052	0.473
AURKA	O14965	0.469
ELN	P15502	0.465
VGFR1	P17948	0.433
CO2A1	P02458	0.430

Table D: The top 10 unobserved targets candidates predicted by Progeni for bronchitis.

Protein Name	Uniprot ID	Prediction Score
IL1R1	P14778	0.395
ALDR	P15121	0.361
TGFB3	P10600	0.343
2AAA	P30153	0.342
NGAL	P80188	0.340
MGST1	P10620	0.339
5NTD	P21589	0.334
TYPH	P19971	0.333
KCNH2	Q12809	0.329
ANT3	P01008	0.327

Table E: The top 10 unobserved targets candidates predicted by Progeni for pulmonary arterial hypertension (PAH).

Protein Name	Uniprot ID	Prediction Score
REG1A	P05451	0.526
CD33	P20138	0.511
HRH4	Q9H3N8	0.487
1A1L1	Q96QU6	0.471
MMP25	Q9NPA2	0.451
SCN2A	Q99250	0.442
IF2G	P41091	0.438
GLP2R	O95838	0.401
KCRU	P12532	0.229
SCNND	P51172	0.226

Table F: The top 10 unobserved targets candidates predicted by Progeni for liver neoplasms.

5 The Trie hashing algorithm for computing C_r values

To efficiently search for the co-occurrences between thousands of pairs of entities in millions of papers from the literature, we adopted the Trie hashing algorithm [14] for substring matching (Algorithm 1).

Algorithm 1 Substring Matching with Trie Hashing

Goal: Record co-occurrence $co[i, j]$ of M entities in N papers.
Collect all M names with at most k characters of the required entities as $entity\_list[M][k]$.
Build Trie Tree:
for all $entity\_name \in entity\_list$ **do**
  $current\_node \leftarrow tree\_root$
  for all $character \in entity\_name$ **do**
    if $current\_node.has\_child(character)$ **then**
      $current\_node \leftarrow current\_node.child(character)$
    else
      $current\_node \leftarrow current\_node.add\_child(character)$
    end if
  end for
  $current\_node.color \leftarrow entity\_index$
end for
Search in N articles:
for all $article \in article\_list$ **do**
  for all $phrase \in article$ **do**
    $current\_node \leftarrow tree\_root$
    for all $character \in phrase$ **do**
      if $current\_node.has\_child(character)$ **then**
        $current\_node \leftarrow current\_node.child(character)$
      else
        $current\_node \leftarrow NULL$
        Break to match the next phrase.
      end if
    end for
    if $current\_node \neq NULL$ **then**
      Record $i \leftarrow current\_node.color$.
      for all recorded entity indices j in this article **do**
        $co[i, j] \leftarrow co[i, j] + 1$
      end for
    end if
  end for
end for

6 Supplementary results on the survival analyses of the top predicted targets in melanoma

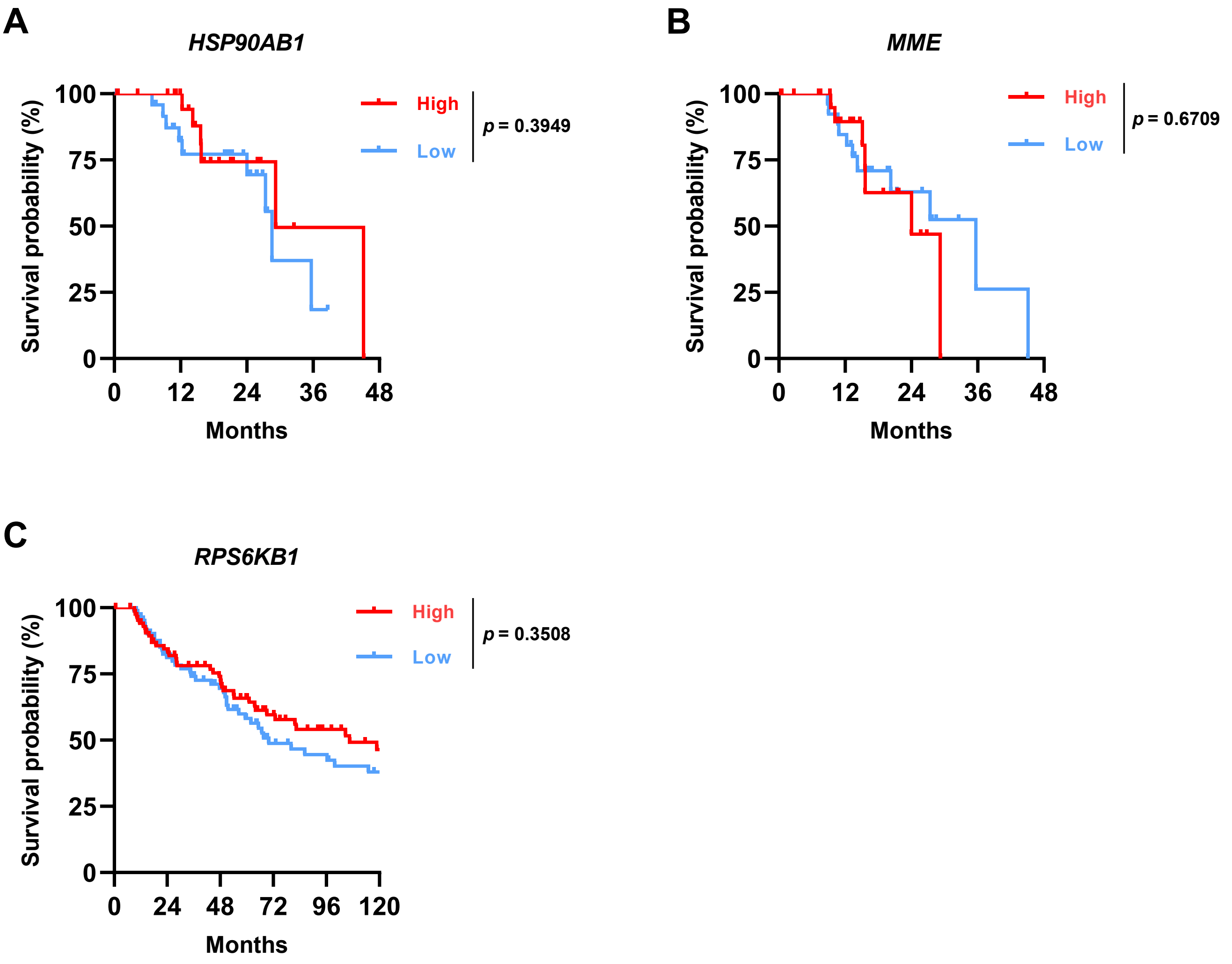

Fig A: (A)-(B), Survival curves of the metastatic melanoma patients from The Cancer Genome Atlas (TCGA) with high or low expression of genes *HSP90AB1* (n=50, **(A)**) and *MME* (n=50, **(B)**), respectively. **(C)**, Survival curves of the primary melanoma patients from TCGA with high or low expression of *RPS6KB1* (n=172).

7 Literature counts for the top 15 novel targets predicted by Progeni for melanoma and colorectal cancer

Gene name	Literature count
CREB1	45
ERBB2	16
HSP90AB1	0
SDCBP	16
CYLD	9
RPS6KB1	0
P2RY12	0
HIF1A	16
TNFSF10	0
ADORA1	1
PIK3R1	4
HLA-A	27
MME	0
HTR1A	0
EEF2	1

Table G: Literature counts for the top 15 novel targets predicted by Progeni for melanoma.

Gene name	Literature count
MDM2	20
ADCY5	0
PAX6	2
MAPK6	0
SLC6A3	0
ADRA2A	0
EEF2	0
GDNF	0
EIF4A3	0
LEP	49
CYP1A1	22
IDH2	2
IL2	0
DLG4	0
JAK2	22

Table H: Literature counts for the top 15 novel targets predicted by Progeni for colorectal cancer.

8 OD values for the CCK-8 assays following shRNA knockdown

In our experiments, all the optical density (OD) values were measured using a BioTek Epoch 2 Microplate Spectrophotometer. The readouts only included an Excel file that recorded all the OD values, as shown below.

Fig 5A				
Backgroud OD value	0.115			
Sample	Control	shHsp90ab1	shRps6kb1	shMme
OD value	1	0.496	0.724	0.467
	1.046	0.502	0.827	0.424
	1.014	0.487	0.751	0.46
	0.955	0.541	0.744	0.471
	0.979	0.514	0.764	0.468
	1.03	0.524	0.792	0.486

Table I: OD values for the B16F10 cells (melanoma) following shRNA knockdown. The results correspond to Fig 5A in the manuscript.

Fig 6A				
Backgroud OD value	0.183			
Sample	Control	shAdcy5	shAdra2a	shEef2
OD value	0.947	0.668	0.75	0.646
	0.907	0.691	0.685	0.647
	0.978	0.724	0.728	0.666
	0.956	0.641	0.734	0.652
	0.982	0.681	0.741	0.647
	1.053	0.701	0.779	0.667

Table J: OD values for the MC38 cells (CRC) following shRNA knockdown. The results correspond to Fig 6A in the manuscript.

9 OD values for the CCK-8 assays after treatment with the small molecule inhibitors

In our experiments, all the optical density (OD) values were measured using a BioTek Epoch 2 Microplate Spectrophotometer. The readouts only included an Excel file that recorded all the OD values, as shown below.

Fig 5E									
Backgroud OD value	0.158								
Drug conc. (nM)	0	1	5	10	50	100	500	1000	5000	10000
OD value	1.446	1.424	1.453	1.514	1.315	1.118	0.721	0.531	0.489	0.479
	1.56	1.466	1.45	1.485	1.352	1.091	0.766	0.546	0.486	0.487
	1.416	1.403	1.461	1.463	1.387	1.181	0.747	0.539	0.489	0.537
	1.492	1.4	1.438	1.491	1.338	1.194	0.769	0.569	0.483	0.487
	1.51	1.616	1.43	1.482	1.342	1.173	0.786	0.573	0.511	0.505

Table K: OD values for the B16F10 cells (melanoma) after 48h treatment with the HSP90AB1 inhibitor tanespimycin. The results correspond to Fig 5E in the manuscript.

Fig 5F										
Backgroud OD value	0.156									
Drug conc. (nM)	0	0.1	0.5	1	5	10	50	100	500	1000
OD value	1.075	1.022	0.993	1.06	1.005	0.969	0.517	0.396	0.363	0.371
	1.087	0.997	1.025	0.98	0.927	0.91	0.474	0.37	0.343	0.361
	1.035	1.009	0.969	1.022	0.978	0.935	0.484	0.385	0.355	0.334
	1.158	0.995	1.017	1.024	0.993	0.853	0.476	0.411	0.377	0.345
	1.112	1.034	1.054	1.013	1.036	1.034	0.506	0.4	0.353	0.347

Table L: OD values for the B16F10 cells (melanoma) after 48h treatment with the HSP90AB1 inhibitor SNX-5422. The results correspond to Fig 5F in the manuscript.

Fig 6E							
Backgroud OD value	0.169						
Drug conc. (μM)	0	0.1	1	5	10	50	100
OD value	1.054	1.014	1.093	1.086	0.93	0.732	0.633
	1.148	1.034	1	1.037	1.04	0.633	0.661
	1.12	1.134	1.053	0.994	0.99	0.649	0.669
	1.232	1.148	1.046	1.12	0.985	0.783	0.736
	1.125	1.151	1.103	1.183	1.027	0.78	0.708

Table M: OD values for the MC38 cells (CRC) after 48h treatment with the ADRA2A inhibitor phentolamine. The results correspond to Fig 6E in the manuscript.

Fig 6F									
Backgroud OD value	0.173								
Drug conc. (μM)	0	0.1	0.5	1	5	10	50	100	500
OD value	0.907	0.816	0.855	0.793	0.804	0.751	0.516	0.372	0.18
	0.808	0.788	0.794	0.842	0.766	0.748	0.523	0.422	0.183
	0.825	0.782	0.791	0.773	0.772	0.764	0.554	0.445	0.186
	0.861	0.819	0.827	0.775	0.787	0.786	0.534	0.427	0.189
	0.915	0.915	0.861	0.88	0.796	0.803	0.512	0.439	0.186

Table N: OD values for the CT26 cells (CRC) after 48h treatment with the ADRA2A inhibitor phentolamine. The results correspond to Fig 6F in the manuscript.

References

[1] Yunan Luo, Xinbin Zhao, Jingtian Zhou, Jinglin Yang, Yanqing Zhang, Wenhua Kuang, Jian Peng, Ligong Chen, and Jianyang Zeng. A network integration approach for drug-target interaction prediction and computational drug repositioning from heterogeneous information. *Nature communications*, 8(1):1–13, 2017.

[2] TS Keshava Prasad, Renu Goel, Kumaran Kandasamy, Shivakumar Keerthikumar, Sameer Kumar, Suresh Mathivanan, Deepthi Telikicherla, Rajesh Raju, Beema Shafreen, Abhilash Venugopal, et al. Human protein reference database—2009 update. *Nucleic acids research*, 37(suppl_1):D767–D772, 2009.

[3] Craig Knox, Vivian Law, Timothy Jewison, Philip Liu, Son Ly, Alex Frolkis, Allison Pon, Kelly Banco, Christine Mak, Vanessa Neveu, et al. Drugbank 3.0: a comprehensive resource for 'omics' research on drugs. *Nucleic acids research*, 39(suppl_1):D1035–D1041, 2010.

[4] Allan Peter Davis, Cynthia J Grondin, Robin J Johnson, Daniela Sciaky, Benjamin L King, Roy McMorran, Jolene Wiegers, Thomas C Wiegers, and Carolyn J Mattingly. The comparative toxicogenomics database: update 2017. *Nucleic acids research*, 45(D1):D972–D978, 2017.

[5] Michael Kuhn, Monica Campillos, Ivica Letunic, Lars Juhl Jensen, and Peer Bork. A side effect resource to capture phenotypic effects of drugs. *Molecular systems biology*, 6(1):343, 2010.

[6] Marinka Zitnik, Monica Agrawal, and Jure Leskovec. Modeling polypharmacy side effects with graph convolutional networks. *Bioinformatics*, 34(13):i457–i466, 2018.

[7] Stanford SNAP Group. Miner: Gigascale multimodal biological network. https://github.com/snap-stanford/miner-data, 2017.

[8] Janet Piñero, Juan Manuel Ramírez-Anguita, Josep Saüch-Pitarch, Francesco Ronzano, Emilio Centeno, Ferran Sanz, and Laura I Furlong. The disgenet knowledge platform for disease genomics: 2019 update. *Nucleic acids research*, 48(D1):D845–D855, 2020.

[9] David S Wishart, Yannick D Feunang, An C Guo, Elvis J Lo, Ana Marcu, Jason R Grant, Tanvir Sajed, Daniel Johnson, Carin Li, Zinat Sayeeda, et al. Drugbank 5.0: a major update to the drugbank database for 2018. *Nucleic acids research*, 46(D1):D1074–D1082, 2018.

[10] Damian Szklarczyk, Alberto Santos, Christian Von Mering, Lars Juhl Jensen, Peer Bork, and Michael Kuhn. Stitch 5: augmenting protein–chemical interaction networks with tissue and affinity data. *Nucleic acids research*, 44(D1):D380–D384, 2016.

[11] Seongjun Yun, Minbyul Jeong, Raehyun Kim, Jaewoo Kang, and Hyunwoo J Kim. Graph transformer networks. *Advances in neural information processing systems*, 32, 2019.

[12] Michael Schlichtkrull, Thomas N Kipf, Peter Bloem, Rianne van den Berg, Ivan Titov, and Max Welling. Modeling relational data with graph convolutional networks. In *European semantic web conference*, pages 593–607. Springer, 2018.

[13] Ziniu Hu, Yuxiao Dong, Kuansan Wang, and Yizhou Sun. Heterogeneous graph transformer. In *Proceedings of The Web Conference 2020*, pages 2704–2710, 2020.

[14] Witold Litwin. Trie hashing. In *Proceedings of the 1981 ACM SIGMOD international conference on Management of data*, pages 19–29, 1981.
