# Supplementary material for: A probabilistic knowledge graph for target identification
Source: PLoS Comput Biol. 2024 Apr 5;20(4):e1011945. doi: 10.1371/journal.pcbi.1011945 (PMC11034645; doi:10.1371/journal.pcbi.1011945)
Supplement: Attachment — Submitted filename: response_Progeni.pdf [file pcbi.1011945.s003.pdf]

Response to the reviewers:

Reviewer #1:

Comment 1: The introduction has a logic red thread through it, however some of the sentences are even for experts hard to understand or too long winded. “On the other hand… and side effects); or “Diffusion-based methods … for specific diseases [15, 12]” are strings of words that contain plenty of jargon that could easily be written shorter or separated into two (or more) sentences. This is prevalent throughout the Introduction, and I would ask this to be changed.

Response:
We thank the reviewer for pointing out this issue. In our revised manuscript, we have broken down every long-winded sentence in the introduction and rephrased many hard-to-read expressions.

Comment 2: In section 2.2 (Results), cross-validation is mentioned: entry-wise and cluster-wise, both fivefold. This is perfectly valid as an internal validation, but Section 4.4 (Methods/Cross-Validation) eludes to the fact that 90% of the folds were used for training. This coincides with the very high performance of all methods, but especially the near-perfect performance of Progeni. None of the numbers are explained in the results section, but I am mostly worried there is a significant case of overtraining happening. This is further strengthened by the mention of the optimisation factoring in during validation. At the end of section 4.3 (Methods/Inference) the authors state that certain terms are added to the optimization objective. While I do trust the authors to have done their due diligence, it is not clear from the text, neither in the results, methods, and the lacklustre discussion.

Response:
We are grateful to the reviewer for pointing out this problem in our manuscript. Cross-validation is a common practice to assess the performance and stability of models. Previous literature [17,26] conducted 10-fold cross-validation together with experimental validation (as external validation) to assess the models. We adopted a similar logic here. Also, we used 5-fold cross-validation, which reduced the training set size to 80% (rather than 90% as in 10-fold cross-validation).

As the reviewer mentioned, Section 4.3 (Methods/Inference) mentioned additional terms in the optimization objective, but their function was unclear. In the revised manuscript, we have explicitly stated that the added regularization terms were meant to combat overfitting to the training set.

Comment 3: To clear these doubts that Progeni is only suitable for the prob-KG dataset,

the authors should try to prove that Progeni is not overfitted/hyperfixated on prob-KG by validating on a new dataset or employing far less data. This section should also include the values of the applied different cross-validations, as well as a section in the discussion comparing the values of the other compared ML methods to what they reported in their original study. Section 2.5 sketches hints of some of these things, including a new dataset, yet the construction of this new dataset and how it feeds into prob-KG is unclear. If any of the former things were shown in the manuscript, then I would ask to clean up the writing and explicitly explain how the datasets feed into prob-KG as described in Figure 1a.

Response:
We thank the reviewer for expressing the concern that Progeni may be overfitted to the prob-KG and that we should emphasize if it is not the case. As the reviewer noted, we manually compiled a new dataset from more up-to-date open sources (Section 2.5). We used this new dataset to predict targets for experimental validation, which we believe is more supportive than purely computational analyses. The curation of the new dataset can be found in the second paragraph of Section 4.1 (Methods/Data Processing).

Since the dataset consists of, mathematically, some weighted adjacency matrices, the way of loading them into the prob-KG/Progeni model, as described in Figure 1a, should be identical to the standard practice of using weighted adjacency matrices as input to GNNs. To make it more readable in the revised manuscript, we have clarified the writing in Section 2.1, which outlines our pipeline.

Comment 4: The discussion unfortunately does not discuss the found results at all, nor does it compare with literature – it is more a summary of the results once more. Especially the fact that established methods have been used for comparison in this manuscript, I expect these methods and results to be compared extensively towards the original studies where these methods were tested.

Response:
We thank the reviewer for stating this problem in our manuscript. We apologize for the lack of discussion in Section 3. We have remedied this by rewriting Section 3, discussing our results in more depth, and retitling it to "Discussion."

Comment 5: The Github documentation was sufficient and clear, though I hope this will be updated once the results are published with an overview on how it works and what the purpose is, for those that want to reproduce the results or generate new ones.

Response:
We appreciate the reviewer for the kind suggestion! We have added an overview to the current GitHub repository that explains the inner workings and purpose of this pipeline.

Reviewer #2:

Major issues:

1. Is there a solid rationale for constructing biological networks using outdated resources? The authors utilized DrugBank v3.0, released in 2011, while the most recent version is 5.1.10, published in 2023. Similarly, they used SIDER v3.0 from 2012 instead of the latest version 4.1, released in 2015. With the significant advancements in these fields over the past decade, the constructed networks and prediction results could vary drastically. It is strongly recommended to use the most recent version.

   Response:
   We thank the reviewer for pointing out this issue. The dataset used for our computational analyses was compiled by the authors of DTINet (Luo et al. 2017, [17]). Since the publication of this paper, numerous other researchers have used this dataset to benchmark model performance in drug discovery applications (e.g., predicting target-drug interactions or target-disease associations). Hence, we would like to use the same widely used dataset for our proof-of-concept, which facilitates benchmarking.

   However, when predicting targets for further experimental validation, we compiled a new dataset from more up-to-date open sources (we explicitly mentioned this feature in Sections 2.5 and 4.1 of the revised manuscript) because we would not want our "novel" predictions to be already present in the literature. We also made this dataset public on GitHub so that future researchers may use this more recent dataset for evaluation purposes.

2. The Comparative Toxicogenomics Database contains curated and inferred drug-disease relationships. It's important for the authors to outline which type was used in their study. For accuracy, it is suggested to limit the usage to curated relationships.

   Response:
   We are thankful to the reviewer for pointing out this concern. In the dataset compiled by Luo et al. used for our computational analyses, a mix of both curated and inferred relationships was used. To facilitate benchmarking, we decided not to change this dataset.

   However, we did take this problem into account when compiling our new dataset to predict novel targets for experimental validation. We mentioned in Section 4.1 (Results/Data Processing) and Supplementary Information Section 1 that we retained only curated relationships.

3. The authors need to provide a rationale for choosing mouse cell lines over human

cell lines for experimental validation, as results from the mouse model may not accurately replicate human outcomes. Furthermore, did the authors limit their calculation of protein-protein co-occurrence frequency to human proteins in the literature? Were orthologs mapped to human proteins? Was mouse protein-protein similarity also considered? To mitigate these potential complications, it is strongly advised to restrict the model to human only.

Response:

We thank the reviewer for noting this issue. We agree that murine cell lines cannot perfectly replicate human diseases and therapy outcomes. However, human cell lines also have this limitation, as natural cancer cells are heterogeneous, while cell lines are much more homogeneous.

Although MC38, CT26, and B16F10 are murine-derived cell lines, they replicate tumor development well, and more importantly, they mimic cancer-immune system interactions. These cell lines have been widely used in pre-clinical research, particularly for evaluating the therapeutic potential of target and drug candidates. Therefore, we believe that these cell lines are sufficient for the experimental validation of targets predicted by Progeni.

4. Co-occurrence might not be the most reliable metric for substantiating protein-disease associations, as the correlation could be opposing, i.e., inhibitory or stimulatory. How are these diverse relation types managed within the probabilistic knowledge graph?

Response:

We are grateful to the reviewers for expressing this doubt. Indeed, as we defined at the beginning of the introduction, a protein can be related to a disease via activation *or* inhibition. That said, we found it difficult to obtain a sufficient amount of data that differentiate between activation and inhibition. Therefore, we chose not to consider these two types of associations separately, but instead only search for a generic relationship between a protein and a disease, regardless of its nature. Such a generic relationship can be reflected by the co-occurrence frequency between a protein and a disease in the literature database, which is easy to obtain and calculate. This approach can easily yield sufficient data for training our machine learning-based model.

5. Only the top-ranked proteins were considered for experimental validation; however, lower-ranked proteins might also be effective in treating melanoma or colorectal cancer. Therefore, it would be advantageous to conduct experiments on some lower-ranked proteins and known treatment targets for melanoma or colorectal cancer as well.

Response:

We thank the reviewer for this suggestion. As we mentioned in our response to the last comment, the rank/output score of the protein would be a measure of "relatedness" or "*absolute value* of correlation" between the protein and a given disease. In this context, low-ranking proteins will have near-0 correlations, and thus have low relevance to the disease. Hence, we did not consider them in our experimental validation.

Minor issues:

1. Initially, I found the term "target" perplexing as it typically refers to a drug target in drug discovery studies. The term "biomarker" might be more intuitive, or alternatively, it may be clearer to refer to it as a "disease target" or "treatment target".

Response:

We appreciate the reviewer for highlighting this issue. We apologize for this confusion. We clarified that our defined "target" has therapeutic potential in "*treating diseases*" in the first sentence of the introduction in our revised manuscript. We also added "disease targets" to the first sentence of the abstract.

2. Which substitution matrix was applied when assessing protein-protein sequence similarity?

Response:

We are grateful to the reviewer for drawing attention to this problem. We have added that we used BLOSUM50 in Section 4.1 (Methods/Data Processing) of the revised manuscript.

3. The arrangement of subfigures (the sequence of a, b, c, and d) in Figure 4 differs from the layout observed in Figures 2 and 3.

Response:

We appreciate the reviewer for noting this inconsistency. We have re-arranged the subfigures in Figure 4 of the revised manuscript to match the configuration of subfigures in other figures in the main text.

4. How is the high or low expression defined within TCGA?

Response:

We thank the reviewer for pointing out this source of confusion. We have added the definitions of high and low-expression cohorts to the captions in Figures 5 and 6 of the revised manuscript.

5. For consistency with Figure 5, it is suggested to present the survival curves for primary colorectal cancer patients from TCGA with high or low expression of ADRA2A and EEF2 in Figure 6, irrespective of the results showing significant differences or not.

Response:

We appreciate the reviewer for drawing attention to this inconsistency. To remedy this problem, we have moved the two survival curves from Supplementary Figure S2 to Figure 6 in our revised manuscript.
