# Supplementary material for: A probabilistic knowledge graph for target identification
Source: PLoS Comput Biol. 2024 Apr 5;20(4):e1011945. doi: 10.1371/journal.pcbi.1011945 (PMC11034645; doi:10.1371/journal.pcbi.1011945)
Supplement: Attachment — Submitted filename: response_Progeni_2.pdf [file pcbi.1011945.s004.pdf]

Response to the reviewers:

Reviewer #1:

Response:

We thank the reviewer for helping us improve our manuscript and approving our modifications.

Reviewer #2:

Major issues:

1. While the authors have updated their datasets, furnished new target candidates, and presented them in the supplementary tables, further validation is lacking. I appreciate the fact that experimental validation may require an extended period of time, but I would encourage the authors to analyze the expressions of these candidates in relation to survival probability at the very least. Furthermore, utilizing the newly added relations in the updated datasets as a test set for the existing model could demonstrate the robustness of the methodology employed.

   Response:

   We thank the reviewer for pointing out this issue. To clarify, as shown in Section 2.5 in our manuscript, we validated target candidates selected from those presented in Supplementary Tables S7 and S8. Specifically, we validated HSP90AB1, RPS6KB1, and MME for melanoma, and ADCY5, ADRA2A, and EEF2 for CRC. We chose these proteins because there is no evidence in the literature suggesting their respective protein-disease associations, whereas the model predicted them as likely target candidates. In our study, we have analyzed their expressions in relation to survival probability and conducted in-vitro experiments (CCK-8 assays with shRNA knockdown or inhibitor treatment) for validation.

2. I concur that utilizing a mouse model is sufficient for the experimental validation of the predicted targets. Nevertheless, there are still certain issues that either remain unresolved or have not been addressed: when determining the frequency of protein-protein co-occurrence and their similarity, were human or mouse proteins employed in the calculations? Were orthologs, not just those of the mouse, but others as well, mapped to human proteins?

   Response:

   We are thankful to the reviewer for pointing out this concern. Throughout the Progeni pipeline (e.g., determining the frequency of disease-protein co-occurrence and protein-protein similarity), we considered only human proteins. Orthologs of

other species were not mapped to human proteins. It is only in the experimental validation that we used mouse cell lines. We have stated this in the revised manuscript.

3. I again recognize that experimental validation could be a lengthy process, however, certain methods such as survival analyses are necessary to justify the relevance of low-ranked proteins. The authors have asserted that "low-ranking proteins will have near-0 correlations, and thus have low relevance to the disease". Without proper validation, this assertion could be questionable since these low-ranking proteins might indeed have significant relevance to the diseases.

Response:
We are grateful to the reviewers for expressing this doubt. We have plotted survival curves for low-ranked targets in both melanoma and colon cancer patients using the TCGA database (refer to Figs R1 and R2). Notably, the low expressions of these low-ranked targets did not confer a survival benefit to patients; in certain instances, it even exacerbated patient survival.

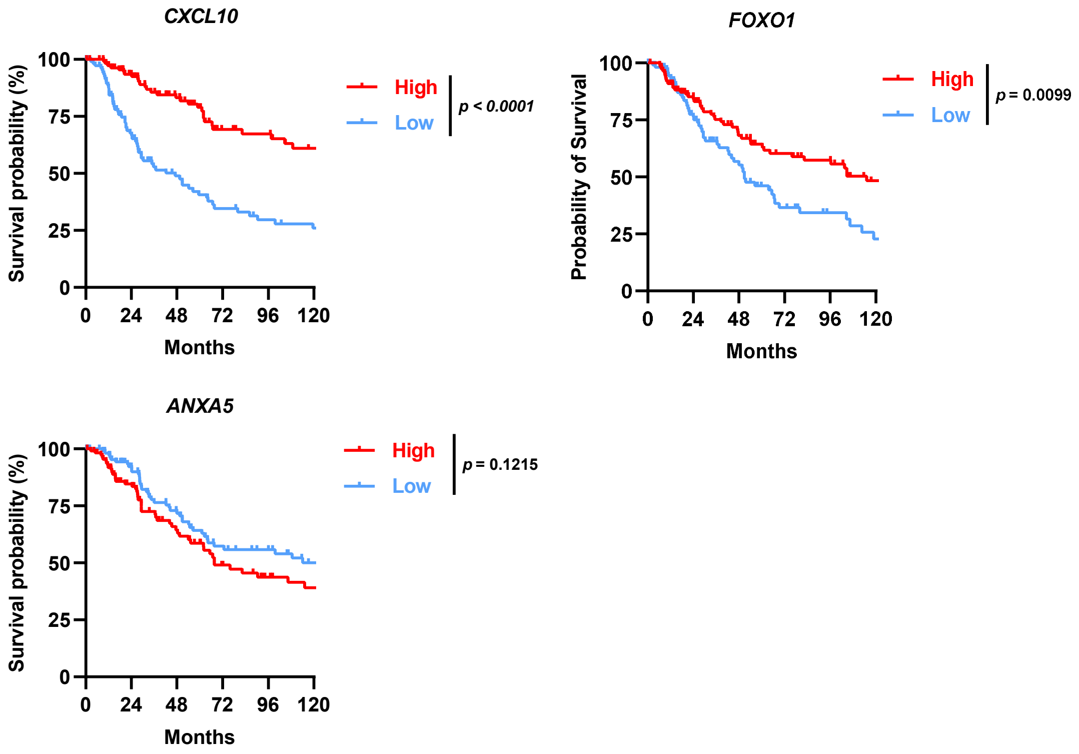

Fig R1. Survival curve of melanoma patients with high or low expression of low-ranked targets (*CXCL10, FOXO1*, and *ANXA5*). The top 25% patients with the highest gene expression were defined as “High,” while the lowest 25% ones were defined as “Low.”

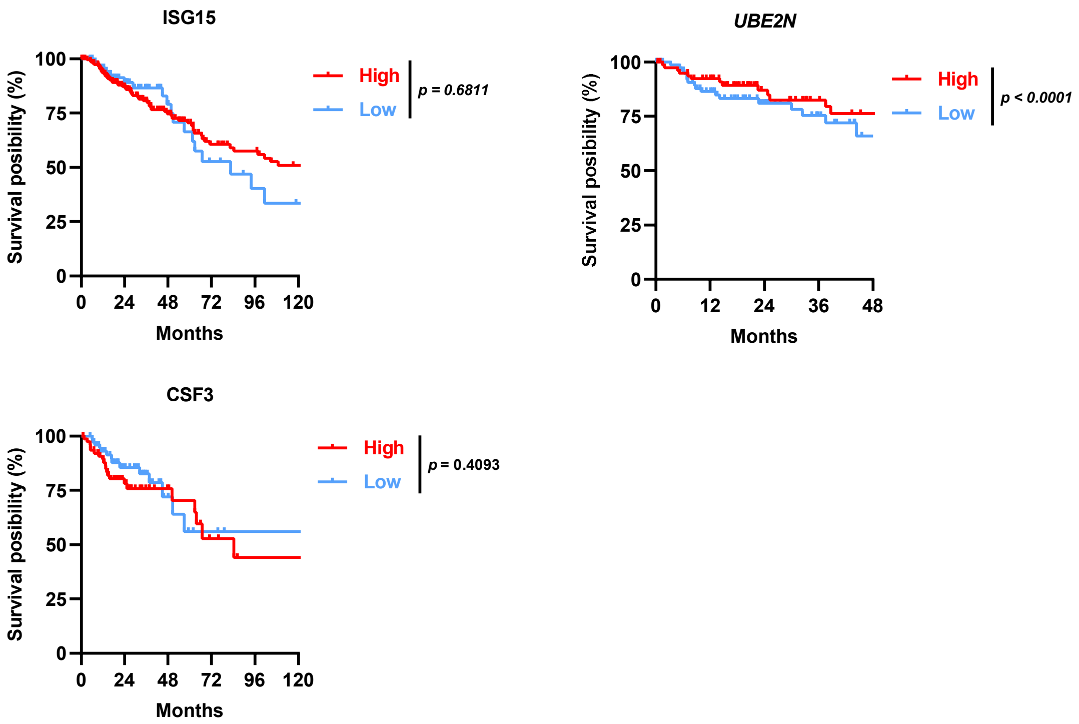

Fig R2. Survival curve of colorectal cancer (CRC) patients with high or low expression of low-ranked targets (*ISG15, UBE2N*, and *CSF3*). The top 25% patients with the highest gene expression were defined as "High," while the lowest 25% ones were defined as "Low."

4. None of the experimental evidence (such as the images of clones) has been attached.

Response:

We thank the reviewer for noting this issue. Our primary method for assessing the viability of cell lines is CCK-8, which gauges cell viability by forming a colored product in living cells. Such colors were recorded as an Optical Density (OD) values. The OD values are generally positively correlated with the numbers of viable cells.

In Tables R1-R6 below, we provide the raw OD values for all the used cell lines following shRNA knockdown or small molecule inhibitor treatment. We hope this information will provide a clearer understanding of the cellular states. In the revised manuscript, we have provided these tables in the Supplementary Information.

Fig 5A				
Backgroud OD value	0.115			
Sample	Control	shHsp90ab1	shRps6kb1	shMme
OD value	1	0.496	0.724	0.467
	1.046	0.502	0.827	0.424
	1.014	0.487	0.751	0.46
	0.955	0.541	0.744	0.471
	0.979	0.514	0.764	0.468
	1.03	0.524	0.792	0.486

Table R1. OD values for the B16F10 cells (melanoma) following shRNA knockdown. The results correspond to Fig 5A in the manuscript.

Fig 6A				
Backgroud OD value	0.183			
Sample	Control	shAdcy5	shAdra2a	shEef2
OD value	0.947	0.668	0.75	0.646
	0.907	0.691	0.685	0.647
	0.978	0.724	0.728	0.666
	0.956	0.641	0.734	0.652
	0.982	0.681	0.741	0.647
	1.053	0.701	0.779	0.667

Table R2. OD values for the MC38 cells (CRC) following shRNA knockdown. The results correspond to Fig 6A in the manuscript.

Fig 5E										
Backgroud OD value	0.158									
Drug conc. (nM)	0	1	5	10	50	100	500	1000	5000	10000
OD value	1.446	1.424	1.453	1.514	1.315	1.118	0.721	0.531	0.489	0.479
	1.56	1.466	1.45	1.485	1.352	1.091	0.766	0.546	0.486	0.487
	1.416	1.403	1.461	1.463	1.387	1.181	0.747	0.539	0.489	0.537
	1.492	1.4	1.438	1.491	1.338	1.194	0.769	0.569	0.483	0.487
	1.51	1.616	1.43	1.482	1.342	1.173	0.786	0.573	0.511	0.505

Table R3. OD values for the B16F10 cells (melanoma) after 48h treatment with the HSP90AB1 inhibitor tanespimycin. The results correspond to Fig 5E in the manuscript.

Fig 5F										
Backgroud OD value	0.156									
Drug conc. (nM)	0	0.1	0.5	1	5	10	50	100	500	1000
OD value	1.075	1.022	0.993	1.06	1.005	0.969	0.517	0.396	0.363	0.371
	1.087	0.997	1.025	0.98	0.927	0.91	0.474	0.37	0.343	0.361
	1.035	1.009	0.969	1.022	0.978	0.935	0.484	0.385	0.355	0.334
	1.158	0.995	1.017	1.024	0.993	0.853	0.476	0.411	0.377	0.345
	1.112	1.034	1.054	1.013	1.036	1.034	0.506	0.4	0.353	0.347

Table R4. OD values for the B16F10 cells (melanoma) after 48h treatment with the HSP90AB1 inhibitor SNX-5422. The results correspond to Fig 5F in the manuscript.

Fig 6E							
Backgroud OD value	0.169						
Drug conc. (μM)	0	0.1	1	5	10	50	100
OD value	1.054	1.014	1.093	1.086	0.93	0.732	0.633
	1.148	1.034	1	1.037	1.04	0.633	0.661
	1.12	1.134	1.053	0.994	0.99	0.649	0.669
	1.232	1.148	1.046	1.12	0.985	0.783	0.736
	1.125	1.151	1.103	1.183	1.027	0.78	0.708

Table R5. OD values for the MC38 cells (CRC) after 48h treatment with the ADRA2A inhibitor phentolamine. The results correspond to Fig 6E in the manuscript.

Fig 6F									
Backgroud OD value	0.173								
Drug conc. (μM)	0	0.1	0.5	1	5	10	50	100	500
OD value	0.907	0.816	0.855	0.793	0.804	0.751	0.516	0.372	0.18
	0.808	0.788	0.794	0.842	0.766	0.748	0.523	0.422	0.183
	0.825	0.782	0.791	0.773	0.772	0.764	0.554	0.445	0.186
	0.861	0.819	0.827	0.775	0.787	0.786	0.534	0.427	0.189
	0.915	0.915	0.861	0.88	0.796	0.803	0.512	0.439	0.186

Table R6. OD values for the CT26 cells (CRC) after 48h treatment with the

ADRA2A inhibitor phentolamine. The results correspond to Fig 6F in the manuscript.

Minor issues:

1. The figures and tables are missing titles and captions.

Response:

We appreciate the reviewer for highlighting this issue. The titles and captions were eliminated from the figure files per the formatting requirements of *PLOS Computational Biology*. The corresponding titles and captions can be found in the manuscript where the figures are to be inserted.
