# Supplementary material for: A probabilistic knowledge graph for target identification
Source: PLoS Comput Biol. 2024 Apr 5;20(4):e1011945. doi: 10.1371/journal.pcbi.1011945 (PMC11034645; doi:10.1371/journal.pcbi.1011945)
Supplement: Attachment — Submitted filename: response_Progeni_3.pdf [file pcbi.1011945.s005.pdf]

Response to the reviewers:

Major issues:

1. The remaining outstanding action item is to provide the experimental images from which the optical density values were taken. These can be added to the supplementary material and will enhance the rigor and reproducibility of the work.

   Response:
   We thank the reviewer for pointing out this issue. To clarify, the optical density (OD) values were measured using a BioTek Epoch 2 Microplate Spectrophotometer. The readouts only included an Excel file that recorded all the OD values; there were no accompanying images. These original readouts were transcribed as evidence in the Supplementary Information (Tables S9-S14).
