# Supplementary material for: A probabilistic knowledge graph for target identification
Source: PLoS Comput Biol. 2024 Apr 5;20(4):e1011945. doi: 10.1371/journal.pcbi.1011945 (PMC11034645; doi:10.1371/journal.pcbi.1011945)
Supplement: Attachment — Submitted filename: PCOMBIOL-D-23-00810 Reviewers comments.docx [file pcbi.1011945.s002.docx]

I reviewed the manuscript ‘A probabilistic knowledge graph for target identification’ by Chang Liu, Kaimin Xiao, Cuinan Yu, Yipin Lei *et al.* The authors set out to use a novel machine learning framework called Progeni, that applies both knowledge graphs as well as predictive capabilities to suggest new targets. They do this by breaking down each step of Progeni and validating the predictions at the end with wetlab experiments. Unfortunately, no line numbers are available so I will try my best to pinpoint to specific parts as much as possible.

The introduction has a logic red thread through it, however some of the sentences are even for experts hard to understand or too long winded. “On the other hand… and side effects); or “Diffusion-based methods … for specific diseases [15, 12]” are strings of words that contain plenty of jargon that could easily be written shorter or separated into two (or more) sentences. This is prevalent throughout the Introduction, and I would ask this to be changed.

The introduction is succinct, and without my comment above would be well suited for this manuscript.

In section 2.2 (Results), cross-validation is mentioned: entry-wise and cluster-wise, both fivefold. This is perfectly valid as an internal validation, but Section 4.4 (Methods/Cross-Validation) eludes to the fact that 90% of the folds were used for training. This coincides with the very high performance of all methods, but especially the near-perfect performance of Progeni. None of the numbers are explained in the results section, but I am mostly worried there is a significant case of overtraining happening. This is further strengthened by the mention of the optimisation factoring in during validation. At the end of section 4.3 (Methods/Inference) the authors state that certain terms are added to the optimization objective. While I do trust the authors to have done their due diligence, it is not clear from the text, neither in the results, methods, and the lacklustre discussion.

To clear these doubts that Progeni is only suitable for the prob-KG dataset, the authors should try to prove that Progeni is not overfitted/hyperfixated on prob-KG by validating on a new dataset or employing far less data. This section should also include the values of the applied different cross-validations, as well as a section in the discussion comparing the values of the other compared ML methods to what they reported in their original study. Section 2.5 sketches hints of some of these things, including a new dataset, yet the construction of this new dataset and how it feeds into prob-KG is unclear. If any of the former things were shown in the manuscript, then I would ask to clean up the writing and explicitly explain how the datasets feed into prob-KG as described in Figure 1a.

I commend the authors for the experimental validation and I am happy to see this has been applied correctly. I enjoyed the graphs as well.

The discussion unfortunately does not discuss the found results at all, nor does it compare with literature – it is more a summary of the results once more. Especially the fact that established methods have been used for comparison in this manuscript, I expect these methods and results to be compared extensively towards the original studies where these methods were tested.

The Github documentation was sufficient and clear, though I hope this will be updated once the results are published with an overview on how it works and what the purpose is, for those that want to reproduce the results or generate new ones.
